# Erratum

**DOI:** 10.1093/jmcb/mjac030

**Published:** 2022-01-28

In the originally published version of the below-listed manuscripts, the html version incorrectly lists the article editor as an author.

These errors have been corrected. The publisher apologizes for the errors.

https://doi.org/10.1093/jmcb/mjab076

https://doi.org/10.1093/jmcb/mjab078

https://doi.org/10.1093/jmcb/mjab079

https://doi.org/10.1093/jmcb/mjab081

https://doi.org/10.1093/jmcb/mjab082

https://doi.org/10.1093/jmcb/mjac002

